# Support needs of patients with COPD: a systematic literature search and narrative review

**DOI:** 10.2147/COPD.S155622

**Published:** 2018-03-26

**Authors:** A Carole Gardener, Gail Ewing, Isla Kuhn, Morag Farquhar

**Affiliations:** 1 Primary Care Unit, Department of Public Health and Primary Care, University of Cambridge, Cambridge, UK; 2 Centre for Family Research, University of Cambridge, Cambridge, UK; 3 University of Cambridge Medical School Library, University of Cambridge, Cambridge, UK; 4 School of Health Sciences, University of East Anglia, Norwich, UK

**Keywords:** COPD, person-centered care, support needs

## Abstract

**Introduction:**

Understanding the breadth of patients’ support needs is important for the delivery of person-centered care, particularly in progressive long-term conditions such as chronic obstructive pulmonary disease (COPD). Existing reviews identify important aspects of managing life with COPD with which patients may need support (support needs); however, none of these comprehensively outlines the full range of support needs that patients can experience. We therefore sought to systematically determine the full range of support needs for patients with COPD to inform development of an evidence-based tool to enable person-centered care.

**Methods:**

We conducted a systematic search and narrative review of the literature. Medline (Ovid), EMBASE, PsycINFO, Cochrane Library, and CINAHL were systematically searched for papers which included data addressing key aspects of support need, as identified by patients with COPD. Relevant data were extracted, and a narrative analysis was conducted.

**Results:**

Thirty-one papers were included in the review, and the following 13 domains (broad areas) of support need were identified: 1) understanding COPD, 2) managing symptoms and medication, 3) healthy lifestyle, 4) managing feelings and worries, 5) living positively with COPD, 6) thinking about the future, 7) anxiety and depression, 8) practical support, 9) finance work and housing, 10) families and close relationships, 11) social and recreational life, 12) independence, and 13) navigating services. These 13 domains of support need were mapped to three of the four overarching categories of need commonly used in relevant national strategy documents (ie, physical, psychological, and social); however, support needs related to the fourth category (spiritual) were notably absent.

**Conclusion:**

This review systematically identifies the comprehensive set of domains of support need for patients with COPD. The findings provide the evidence base for a tool to help patients identify and express their support needs, which underpins a proposed intervention to enable the delivery of person-centered care: the Support Needs Approach for Patients (SNAP).

## Introduction

Patients in the advanced stage of chronic obstructive pulmonary disease (COPD) can experience a range of debilitating physical symptoms, resulting in a loss of functionality and high levels of psychosocial distress.^1^–^4^ National strategy documents^5^,^6^ highlight the need to address individual physical, psychological, social, and spiritual needs experienced by these patients through holistic supportive input delivered through person-centered care. Understanding the patient’s view on their support needs (those aspects of managing life with COPD with which they need support, eg, support to manage their symptoms or access financial benefits) is key to facilitating this approach.

Existing reviews addressing patient perspectives on need in advanced COPD have tended to be focused, for example, on patient difficulties (eg, breathlessness^7^–^9^ and isolation^7^–^10^) or on patient requirements for specific aspects of supportive input (eg, information^8^,^9^ and nursing care^8^,^9^). The range of underlying support needs identified by these reviews is therefore limited. Disler et al^10^ and Spathis and Booth^7^ highlighted patients’ need for support in understanding COPD and knowing what to expect in the future, while Gardiner et al^8^ identified a need for assistance in managing personal care and practical tasks. Patient need for support in relation to accessing aides and adaptions, getting out and about, managing feelings of isolations and depression, and claiming financial benefits have also been reported.^7^–^10^ However, none of these existing reviews comprehensively outlines the full range of support needs patients can experience, limiting our ability to develop evidence-based interventions to identify and address patients’ unmet support needs in advanced COPD. Thus, we sought to identify key aspects of support need identified by patients via a systematic review of the relevant literature in order to determine the full range of support needs for patients with COPD. This review underpins a program of work to develop a designed-for-purpose, evidence-based tool to help patients with advanced COPD identify and express their support needs with health care professionals, in order to enable a needs-led conversation, facilitating person-centered care.

## Methods

We conducted a systematic review of the literature to identify key aspects of support need in patients with advanced COPD. The search followed the principles of a systematic review, with reference to Preferred Reporting Items for Systematic Reviews and Meta-Analyses (PRISMA) guidelines,^11^ incorporating limiters and adopting a pragmatic approach to the assessment of studies for inclusion in order to expedite delivery. The four authors brought a range of perspectives to the review including palliative care research (MF, GE, and ACG), nursing (MF and GE), social work (ACG), and information specialism (IK).

### Inclusion criteria

The inclusion criteria are outlined in Table 1.

#### Types of participants

The review considered all studies that involved human subjects who were adult (18 years and older) and diagnosed with COPD. The inclusion of studies relating to patients with COPD, rather than just advanced COPD, followed an initial scoping of the literature which identified a limited number of studies specifically addressing patients’ support needs in advanced disease.

#### Support needs

Studies were included if they addressed key aspects of support need, as identified by the patient. In conducting similar work in relation to carers, Ewing and Grande^12^ described the following three types of data that may indicate support needs: 1) carer support needs that were met, 2) supportive input that was perceived as helpful by carers, and 3) shortfalls in provision where carer needs had not been met. These three types of data were used to guide a framework for the identification of support needs to enable the identification of relevant papers (and data) for this present review: 1) patient support needs that were met (met needs), 2) supportive input that was perceived as helpful by patients (helpful input), and 3) shortfalls in provision where patients’ needs had not been met (unmet needs).

#### Types of studies

The review considered studies that included patient perspectives on support need through quantitative, qualitative, or mixed methods research designs.

### Search strategy

The search strategy, developed with our information specialist (IK), comprised the following three stages:

1. Pilot search: an initial search of Medline Ovid was undertaken using keywords and phrases from key articles in the subject area. The terms “COPD” and “need” were found to be the most effective in identifying relevant material.
2. Extended electronic search informed by the pilot search: search terms used are shown in Table 2. For pragmatic reasons within this review, search terms were limited to abstract and title only. The search terms were then applied to each of the following electronic databases: Medline (Ovid), EMBASE, PsycINFO, Cochrane Library, and CINAHL. Studies had to be primary research and published in peer review journals. Given the pragmatic nature of the review only studies written in English and published in the previous 20 years (January 1996 to February 2016) were considered.
3. Manual search: reference lists of relevant systematic reviews identified through the search were checked for further potentially relevant papers based on their titles or commentaries within reviews.

**Figure 1:**
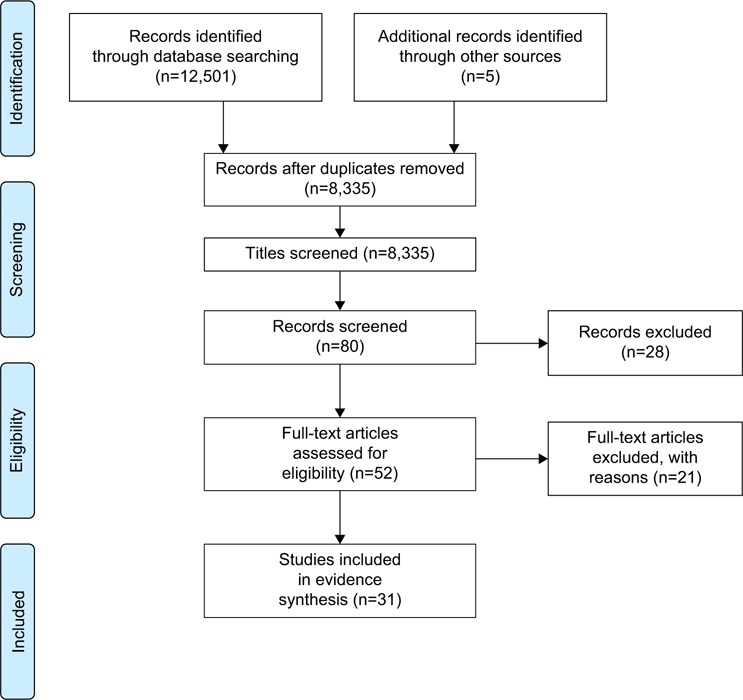
PRISMA flow diagram. **Abbreviation:** PRISMA, Preferred Reporting Items for Systematic Reviews and Meta-Analyses.

The three stages of the search strategy comprise the identification step referred to on the PRISMA flow diagram (Figure 1) that summarizes the systematic review process.

### Abstract selection procedure

Titles and abstracts of studies to be considered for retrieval were recorded in an EndNote database along with details of where the reference was found. Titles and abstracts were screened by the lead reviewer (ACG) and those that clearly did not meet the inclusion criteria were excluded by the lead reviewer only.

Two reviewers (ACG/MF) independently reviewed the remaining abstracts. Abstracts were assessed for their relevance to the topic, using the framework for the identification of support needs adopted by the review, and outlined earlier: met needs, helpful input, and unmet needs. Discrepancies in the selection process were resolved by discussion prior to data extraction.

### Assessment of study quality

Full copies of articles identified as potentially relevant were obtained and assessed for methodological quality by the lead reviewer (ACG). We used the following five-category rating of Dixon-Woods et al^13^ to assess study quality using unprompted judgment: KP – key paper to be included in review, SP – satisfactory paper to be included in review, ? – unsure whether paper should be included, FF – paper to be excluded on the grounds of being fatally flawed, and IRR – paper to be excluded in the grounds that it is irrelevant.

### Data extraction and synthesis

#### Stage 1 (extraction)

Data relating to each of the three types of data in the adopted framework for the identification of support needs were extracted from the included papers onto an Excel spreadsheet, using narrative analysis, by the lead reviewer. A random sample of these papers was also analyzed by the second reviewer (MF), and any disagreements on categorization resolved through discussion.

#### Stage 2 (synthesis)

Areas of support need that emerged from the extracted data were subsequently reviewed and revised through team discussion (ACG, MF, and GE). To facilitate presentation, these areas of support need were then mapped to the following four overarching categories of need informed by the national framework document “Ambitions for Palliative and End of Life Care”:^5^ 1) physical, 2) psychological, 3) social, and 4) spiritual.

## Results

The results are presented in two sections: first, details of included studies are summarized and, second, identified areas of support need are presented within the four overarching categories of need.

### Section 1: overview of included studies

The PRISMA flow diagram (Figure 1) summarized the systematic review outcome. Details of the papers included are outlined in Table 3. Thirty-one papers were included in the review. Most of the papers were from the UK (n=16), followed by Canada (n=4) with three papers each from the USA, Sweden, and Australia and one paper from Norway. One further paper related to a study conducted across five European countries (UK, Germany, France, Italy, and Spain). Twenty-seven papers described studies using qualitative methodologies and four papers used a mixed-methods approach. Together the papers reported on the perspectives of patients with COPD across a range of topics and settings; however, all papers included data related to patient-identified support needs.

### Section 2: areas of support need organized by overarching categories of need and detailed description

The following 13 broad areas of support need (referred to as domains) were identified: 1) understanding COPD, 2) managing symptoms and medication, 3) healthy lifestyle, 4) managing feelings and worries, 5) living positively with COPD, 6) thinking about the future, 7) anxiety and depression, 8) practical support, 9) finance work and housing, 10) families and close relationships, 11) social and recreational life, 12) independence, and 13) navigating services. A detailed description of each is provided below, presented within the four overarching categories of need (physical, psychological, social, and spiritual).

Table 4 provides an overview of the 13 identified domains mapped to met needs, supportive inputs, and unmet needs as reported in the literature.

#### Physical

The majority of studies included in the review identified support needs in relation to patients’ physical health.

##### Understanding COPD

Fourteen studies identified patients’ understanding of the nature of COPD as a key aspect of support, with an important issue being the lack of information provided about their condition.^14^–^27^ When they were given information, this was perceived by patients as highly beneficial.^14^,^15^,^17^ A number of studies identified the need for understanding the term COPD,^16^,^22^ understanding the nature of lung damage associated with COPD^15^,^25^ and greater clarity about COPD at the time of diagnosis.^16^,^18^,^19^,^27^

Twelve studies identified the desire of some patients to be made aware of how their symptoms would progress in the future and their likely prognosis.^16^,^18^,^19^,^21^,^25^,^26^,^28^–^33^ While it was acknowledged that not all patients with COPD wanted to be made aware of the future, it was clear that for many there was a need to understand how long they had to live^18^,^31^,^32^ and the likely nature of their symptoms at the advanced stage of illness.^19^,^26^,^28^,^31^

##### Managing symptoms and medication

Eleven studies identified patients’ need for support with managing their condition. These studies described how patients valued, or identified, a need for basic information and instruction in relation to 1) coping with symptoms^14^–^17^,^24^,^26^ (eg, pacing, dealing with panic attacks, and breathing exercises); 2) treatment options^20^,^25^,^29^ and effective use of medication^14^,^20^,^22^,^24^–^26^ (eg, correct inhaler technique, when to use standby packs, and nebulizers); and 3) the provision and use of oxygen.^34^

A number of the studies also reported patients’ need for a more dynamic form of support, particularly in the context of managing exacerbations, emergencies, admissions, and discharge. For example, patients valued guidance as to when to take standby medication or when to go to hospital,^20^,^21^,^24^,^32^,^34^ monitoring and feedback about whether or not they were assessing and managing a situation correctly,^17^,^30^,^32^,^34^,^35^ and at times, having someone else to take over the responsibility for decision making.^15^,^32^,^36^ Studies also noted the value placed on having easy access to both health care professionals and family members who could be contacted when patients were worried or needed assistance.^14^,^15^,^32^,^34^,^37^

##### Healthy lifestyle

Six studies highlighted a need for support in relation to how patients could lead a healthier lifestyle.^17^,^20^,^22^,^24^,^38^,^39^ Three of the studies identified patients’ need for support around exercise and activity in the following areas:^17^,^22^,^24^ 1) encouragement to exercise,^17^,^22^ 2) exercising safely,^22^ 3) identifying and achieving personal activity goals,^17^ and 4) developing the ability and confidence to use exercise equipment at home.^24^ Two studies focused on patient support in the context of smoking cessation.^38^,^39^ Both studies acknowledged the difficulties involved in stopping smoking but reported patients’ views on key aspects of support patients had found useful or would have welcomed. Patients highlighted the importance of hearing the right words of encouragement at the right time, ongoing praise and encouragement, being made aware of available cessation services, and accessing immediate support when necessary. Three of the studies highlighted a need not to feel blamed for current or previous lifestyle choices.^20^,^38^,^39^

#### Psychological and emotional

The reported need for emotional support by patients with COPD is well documented;^8^,^35^,^37^ however, few authors have focused on identifying in any detail what patients actually need in order to feel emotionally supported. However, the following four key areas of psychological support did emerge from the review: managing feelings and worries, living positively with COPD, thinking about the future, and anxiety and depression, each of which are discussed below.

##### Managing feelings and worries

In their study of patients’ experiences in a hospice setting, Hayle et al^40^ highlighted the difficulties in pinpointing factors that contribute to the enhanced psychological well-being of patients but reported the importance patients themselves place on the awareness of being cared for and the opportunity for the honest expression of emotions. Patients described the confidence and sense of self-worth that comes from being able to share and discuss their feelings, together with the opportunity provided to overcome more distressing feelings. Other studies have drawn attention to the value patients place on interactions in which they feel listened to, perceive empathy and understanding from others, and have the opportunity to discuss how they are feeling.^14^,^15^,^20^,^22^ Patients also frequently describe the positive impact on their emotional well-being when they are taken seriously and feel that they are being seen as an individual.^27^,^35^,^40^

##### Living positively with COPD

Five studies considered support in relation to the emotional adjustment patients may need to make in order to live with COPD.^15^,^17^,^22^,^27^,^35^ In relation to talking to understanding others or experience of accessing peer support, these studies highlight key needs that are addressed by such support: knowing you are not alone in having COPD,^27^,^35^ sharing and validating experiences,^27^,^35^ letting go of criticism and self-blame,^27^ and being able to draw on others for encouragement, advice, and strategies to support living in a positive way.^15^,^17^,^22^,^35^

##### Thinking about the future

In addition to the need for information, noted in the “Understanding COPD” section, two studies highlighted the need to address the emotions surrounding end of life: patients noted, in particular, the value in observing and talking to other patients who were also living with life-limiting conditions.^31^,^40^ They reported this helped them to keep end of life issues in perspective and feel more optimistic about the future. As noted in the “Understanding COPD” section, not all patients are comfortable with discussing end of life issues; however, for others, it was important to discuss disease progression and prognosis so they could plan for their future care needs, symptom management, and practical considerations.^19^,^21^,^26^,^31^,^33^ Schroedl et al^28^ found that some patients reported comfort in having made plans for death.

##### Anxiety and depression

Despite the high prevalence of anxiety and depression reported within this patient group,^41^ there is surprisingly little discussion in the literature, beyond the areas discussed earlier, about particular support needs for those with psychological co-morbidities. Ellison et al^15^ noted a reluctance by study participants to access specialist interventions to manage psychological symptoms, such as medication. In contrast, the value of “talking therapies” was highlighted in three studies.^15^,^22^,^24^

#### Social

The need for support in relation to social issues covers a broad spectrum of difficulties faced by patients with COPD as outlined later.

##### Practical support

In the context of managing roles both at home and in the community, there is clear evidence of patients’ need for practical support in the following three key areas: personal care, managing the home and garden, and mobility.^30^,^32^,^36^,^37^,^42^–^44^ Ek et al,^36^ Odencrants et al,^43^ and Jackson et al^37^ describe how patients frequently rely on support with personal hygiene, cooking, shopping, cleaning, transport, and other strenuous household tasks in some detail.

##### Finance, legal issues, and housing

None of the studies reported patients directly stating a need for support with their finances; however, many patients with COPD claim some form of welfare benefits. There was evidence of patient frustration and concerns over the lack of information and the time required to access financial benefits.^16^,^30^,^34^,^45^ Being made aware of potential financial benefits was seen as one of the advantages of attending pulmonary rehabilitation.^22^ In a Canadian study, Jackson et al^37^ noted that financial benefits were critical for those accessing health care and Lindgren et al^27^ concluded that access to welfare benefits increased patients’ ability to live in the best way possible. In addition to finances, there was evidence of patient support need in relation to exploring housing options,^21^,^34^ and in the context of future planning, the evidence highlights how housing, financial, and legal issues are also important components of this support domain.^21^,^26^,^31^

##### Families and close relationships

The importance patients place on family and close relationships was reported in a number of studies, together with concern about family anxiety and potential strain on relationships when family and friends take on caring roles.^14^,^42^ However, there was little discussion by patients of how these relationships could be supported, with just one study drawing attention to carer need for a better understanding of COPD and a further study suggesting that there were mixed views on whether carer support was necessary.^22^,^30^

##### Social and recreational life

The value placed by patients on opportunities for social interaction with families and friends underlines the need for support in the face of isolation and loneliness.^37^,^42^ Guthrie et al^42^ evidenced a need for both practical support to maintain existing relationships and interests (eg, transport) and the need for opportunities to develop new support structures. Studies by Gysels and Higginson,^17^ Rodgers et al,^22^ and Hayle et al^40^ considered the nature of social support obtained from attending pulmonary rehabilitation or a day hospice. Benefits of these programs included encouragement to get out of the house, the chance to meet people, the opportunity to make friends, and being able to enjoy the company of others.

##### Independence

While for some patients the need for support inevitably resulted in increasing dependency on others, a number of studies drew attention to aspects of support that enable patients to maximize their independence and engagement in the wider community.^33^,^36^,^42^ Ek et al^36^ and Guthrie et al^42^ highlighted how support related to independence is frequently understood in terms of accessing equipment and assistive devices. Patients typically used, or expressed a need for, chair-lifts, equipment to help with food preparation, bathroom aids, mobility scooters, and cars.^16^,^32^,^36^,^42^,^43^,^45^

Guthrie et al^42^ and Ek et al^36^ concluded that the need for support in maintaining independence through mobility is a key issue. Guthrie et al argued that car ownership provided a “distinct advantage in supporting patients in keeping up leisure pursuits and shopping”,^42^ and Ek et al^36^ noted that patients with a mobility scooter had more opportunity to get out of the house thereby expanding both their living space and opportunities for social interaction. A related support need is better understanding about the availability of these resources and assistance to access these resources.^16^,^32^,^45^,^46^

#### Navigating services

Living with a long-term condition frequently involves navigating a complex system of service providers, appointments, and information with which patients can at times need support.^37^ As with physical needs, a key theme in the literature is a need for understanding and awareness of the services that could potentially support patients both now and in the future.^16^,^18^,^21^,^34^ Gruffydd-Jones et al^34^ and Gore et al^16^ highlighted patients’ need for a greater awareness of available benefits, housing, and possible options for treatment and care. Gysels and Higginson^18^ documented the additional difficulties that patients can experience gaining access to services. Jackson et al^37^ report that, even when patients are in contact with service providers, there can still be a continued need for support to make appointments, process information, put forward views, and facilitate a positive working relationship with health care professionals. They also noted that the presence of family and friends was considered to enhance the quality of interactions with health care systems.

#### Spiritual

None of the papers reported support needs that could be directly related to religious considerations, eg, support to access religious services or items, or to ensure that religious requirements and restrictions were observed. With the exception of Hayle et al,^40^ the papers reviewed did not specifically address existential or value-based needs, although related concerns such as issues of loss and dealing with feelings and worries relating to end of life were raised under psychological needs.

## Discussion

In this review, we sought to systematically determine the comprehensive set of domains of support need for patients with COPD. The review identified wide ranging areas of support that patients need, or value, in order to manage life with COPD. This extracted evidence was synthesized and formulated into 13 domains of patient support need.

We identified 31 papers that included data on support needs, as described by patients with COPD. The focus on papers incorporating patients’ views (rather than the views of health care professionals or carers) was adopted to meet the requirements of a wider program of work, underpinned by this review, to develop a designed-for-purpose tool to enable patients with advanced COPD to identify and express their support needs. Although in this context, it might have been expected that terms such as “felt need” and “expressed need” deriving from Bradshaw’s taxonomy^47^ would have been key search terms, it is of note methodologically that, in order to identify relevant papers, the search terms “need” or “needs” were found to generate more relevant studies reporting patients’ perspectives of support need. Relevant papers included those explicitly investigating the needs of patients with COPD and those exploring patients’ experiences of living with COPD.

The data were extracted using a framework that enabled a very inclusive approach to identify a full range of support needs: patients’ support needs that were met (met needs), supportive input that was perceived helpful by patients (supportive input), and shortfalls in provision where patients’ needs had not been met (unmet needs). Synthesizing the extracted data into the domains was generally straightforward. On some occasions, data were found to relate to more than one domain of need, for example, key aspects of support within the domain “finance, work, and housing” were also relevant to the domain “thinking about the future”. Similarly, there was some debate among the review team regarding how to map some of the support needs to the four categories, eg, whether personal care should be considered as social or medical. These differing views reflected the various disciplines represented within the review team and were resolved through discussion, with the focus less on which category was the most appropriate and more on whether the categorization was pertinent from both a medical and social care perspective. Other challenges arose when papers described support in very general terms. One example was the use of the term “emotional support” that was often used without describing what patients actually needed in order to feel emotionally supported. Emotional support was also described in relation to the networks that patients build with their families, peers, and health care professionals. However, as these relationships frequently crossed the boundaries of emotional, social, and practical supports, it was again unclear which aspects of these relationships patients valued in relation to their emotional needs.

The findings from this review build on our understanding of patients’ support needs in a number of different ways. First, it moves the literature beyond existing reviews, which predominantly provide accounts of “indicators of need” to conceptualizing support domains, which can be used to identify directly the support needs of patients. Reviews, which identify the impact of living with COPD (eg, depression and loss of functionality), are in themselves very valuable but in so doing highlight only areas indirectly indicating that a patient may need support: they do not identify what they need help with. By focusing on those areas in which patients report directly that they need support, this review provides an alternative approach with potential to enable health care professionals to better understand patient need, as well as supporting patients in identifying and expressing those needs.

The review further adds to the understanding of patients’ support needs in COPD by providing a clear evidence base for a comprehensive set of domains of support need. Through summarizing reviews and individual studies, the synthesis of evidence into 13 broad areas of support need has identified a range of additional areas in which patients say they need assistance, for example, exercising safely, navigating services, and overcoming feelings of guilt. These resonate with the physical, psychological, and social support needs of patients with other advanced diseases.^48^,^49^ However, it is noteworthy that none of the studies reviewed reported patients’ need for support in relation to spirituality, which has been shown to be important in end of life care for conditions such as cancer.^50^ This may reflect the differing nature of the course of COPD in comparison to malignant conditions in which people may perceive of themselves as living with, rather than dying from, COPD and has implications for the application of “one size fits all” guidelines.^51^

The identification of a comprehensive set of domains of support need for patients with COPD also has significance for the delivery of supportive, palliative, and end of life care. The current focus is on using the House of Care Model^52^ to deliver a person-centered approach to support people with long-term conditions. This model seeks to engage patients to best manage their condition. Yet, we know that patients have difficulty in articulating their needs.^53^ The comprehensive set of support domains identified by this review offers a potential framework to provide visibility of relevant broad areas of support, thus enabling patients to express their needs within the existing House of Care Model. An intervention based on this comprehensive set of support domains is currently undergoing validation to enable the delivery of person-centered care: the Support Needs Approach for Patients (SNAP).^54^

Potential limitations to this review include the exclusion of papers published outside the time frame 1996–2016, which may have resulted in aspects of support being overlooked if reported earlier. The broad search terms used in the review allowed the inclusion of a range of qualitative studies in which aspects of support were discussed by patients; however, it is possible that there are qualitative studies with a different emphasis, which also cover areas of support need that were not included. In addition, the known difficulty patients with COPD have in articulating their needs could, in itself, have influenced the range of support needs identified within the studies included in the review; however, this difficulty predominantly relates to clinical contexts.

## Conclusion

This review systematically identifies the comprehensive set of domains of support need for patients with COPD, using the perspectives of those best placed to identify them: the patients themselves. These findings have implications for practice, enabling clinicians to enhance patient support, and for research by providing an evidence base for an intervention to assess the support needs of patients using a person-centered approach. Finally, this review has made clear that there are commonalities but also differences in the situations of patients with COPD and their resultant support needs compared with other life-limiting conditions such as cancer. As such it contributes to the current “refreshing” of the End of Life Care Strategy as the Ambitions for Palliative and End of Life Care,^5^ which more fully integrates long-term conditions.

## Figures and Tables

**Table 1:** Inclusion criteria

1. Some or all of the participants are patients with COPD
2. Adults (18 years +)
3. Paper includes data identifying support needs in patients with COPD
4. The support needs are identified by patients with COPD
5. Peer reviewed journal
6. Primary research paper
7. English language

**Abbreviation:** COPD, chronic obstructive pulmonary disease.

**Table 2:** Medline (Ovid) search strategy

1. (COPD or chronic obstructive pulmonary disease).mp.
2. exp Pulmonary Disease, Chronic Obstructive/
3. (need or needs).mp. [mp=title, abstract, original title, name of substance word, subject heading word, keyword heading word, protocol supplementary concept word, rare disease supplementary concept word, unique identifier, synonyms] (990320)
4. 1 or 2
5. 3 and 4
Date limiters: 1996–2016

**Abbreviation:** COPD, chronic obstructive pulmonary disease.

**Table 3:** Characteristics of included studies

Reference (year), country	Recruitment setting	Sample size	Severity of COPD	Participant characteristics	Methods	Analysis
Booth et al (2003)^14^ UK	Respiratory clinical within a university teaching hospital	10	3 or 4 on modified MRC 4-point scale	Male =6 Age range =51–80 years	Semistructured interviews	Qualitative: thematic
Cicutto et al (2004)^35^ Canada	Community	42	Physician diagnosed COPD Daily symptoms that limit activities	Male =55% Age range =54–74 years	Focus groups	Qualitative: constant comparative
Ek et al (2011)^36^ Sweden	Pulmonary specialist clinic	4	COPD as primary diagnosis LTOT (16–18 hours a day)	Male =1 Age range =54–71 years	Longitudinal qualitative interviews	Qualitative: phenomenological hermeneutical
Ellison et al (2012)^15^ UK	Community-based COPD outpatient clinic	14	Spirometry confirmed diagnosis of COPD	Male =7 Age range =49–79 years	In-depth semistructured interviews	Qualitative: constant comparative
Gore et al (2000)^16^ UK	Chest clinic	50	FEV_1_ <0.751 At least one admission for hypercapnic respiratory failure	Male =44% Mean age =70.5 years	Semistructured interviews Quality of life tools Document review	Qualitative: thematic
Gullick and Stainton (2006)^38^ Australia	Three teaching hospitals	15	Severe emphysema FEV_1_ range 15%–51%	Male =9 Age range =55–77 years	Semistructured interviews	Qualitative: hermeneutic phenomenology
Guthrie et al (2001)^42^ UK	Not reported (sample taken from patients participating in a larger study)	37 (20 at second interview)	Severe COPD	Male =8 Mean age =67 years (sample of 20)	Longitudinal semistructured interviews	Qualitative
Gruffydd-Jones et al (2007)^34^ UK	District general hospital	25	Postadmission with a diagnosis of an acute exacerbation of COPD	Male =11 Mean age =76 years	Standardized measures Hospital records Semistructured interviews Focus groups	Quantitative: descriptive statistics Qualitative: interpretive phenomenology
Gysels and Higginson (2009)^17^ UK	Hospital respiratory clinics, specialist respiratory nurses’ rounds and consultations, “Breathe Easy” service user meetings, and a GP practice disease register	18	Diagnosis of COPD Daily problems of breathlessness	Male =7 Age range =52–78 years	Participant observation In-depth interviews	Qualitative: grounded theory
Gysels and Higginson (2010)^18^ UK	Hospital respiratory clinics, specialist respiratory nurses’ rounds and consultations, Breathe Easy service user meetings, and a GP practice disease	18	Breathlessness as a problematic symptom of COPD	Male =7 Median age: Outpatients =69 years Community =70 years	Participant observation In-depth interviews	Qualitative: narrative
Hayle et al (2013)^40^ UK	NHS Trust and independent hospice	8	Primary diagnosis of COPD Accessing specialist palliative care	Male =5 Age range =63–77 years	Semistructured interviews	Qualitative: hermeneutic phenomenology
Hasson et al (2008)^30^ UK	Hospital	13	FEV_1_ <30% or LTOT or noninvasive ventilation Optimal drug therapy	Male =10 Median age =65 years	Semistructured interviews	Qualitative
Jackson et al (2012)^37^ Canada	Acute care nursing unit during admission	4	Diagnosis of COPD	Male =1 Age range =57–58 years	Multiple case study methods	Qualitative: thematic
Jones et al (2004)^32^ UK	Primary care practices	16	Maximal therapy for COPD Considered to be in last year of life	Male =8 Age range =62–83 years	Semistructured interviews	Qualitative: thematic
Lindgren et al (2014)^27^ Norway	Three GP practices and an outpatient pulmonary rehabilitation clinic	8	Diagnosed with mild or moderate COPD	Male =3 Age range =60–74 years	Semistructured interviews	Qualitative: phenomenological hermeneutic
Lowey et al (2013)^33^ USA	Two Medicare-certified home health agencies	10	Oxygen-dependent COPD	Not reported	Semistructured interviews	Qualitative: thematic
MacPherson et al (2013)^19^ UK	GP practice and hospital respiratory team	10	Severe COPD (Gold Standards Framework criteria)	Male =9 Age range =58–86 years	Semistructured interviews	Qualitative: grounded theory
McDonald et al (2013)^25^ Australia	Hospital-based respiratory ambulatory care clinics based	7	Confirmed diagnosis of COPD FEV_1_% predicted mean =44	Male =3 Mean age =68.7 years	In-depth semistructured interviews	Qualitative: thematic
Nykvist et al (2014)^39^ Sweden	Primary care	6	Diagnosis of COPD	Female =6 Age not reported	Narrative interviews	Qualitative: narrative
Odencrants et al (2005)^43^ Sweden	Five primary health care clinics	13	A diagnosis of COPD according to ICD-10 FEV_1_ <50%	Male =5 Mean age =68.9 years	Self-reported diary Semistructured interviews	Qualitative: content
Oliver (2001)^24^ UK	One GP practice and a district general hospital	17	Diagnosis of COPD FEV_1_ <50% of predicted value	Male =12 Age range =59–75 years	Semistructured interviews	Qualitative: thematic
Partridge et al (2011)^20^ UK, France Italy, Spain Germany	Prerecruited panel who had agreed to take part in research opinion studies	719	MRC score >3	Male (%) =30.5 Mean age =62.4 years	Quantitative questionnaire- based survey	Quantitative
Philip et al (2012)^21^ Australia	Respiratory outpatient hospital in a tertiary hospital	10	COPD Recent admission for a life-threatening exacerbation	Male =6 Age range =55–76 years	In-depth semistructured interviews	Qualitative: thematic
Rodgers et al (2007)^22^ UK	Pulmonary rehabilitation program within a community hospital	23	COPD patients who had attended pulmonary rehabilitation	Male =14 Mean age for each focus group (×4): 65, 68, 63, 70	Focus groups	Qualitative: template
Schroedl et al (2014)^28^ USA	Academic medical center	20	History of COPD Hospital admission following exacerbation	Male =9 Age range =52–83 years	Semistructured interviews	Qualitative: thematic
Seamark et al (2004)^29^ UK	GP practice	10	Diagnosis of COPD FEV_1_ <40% predicted	Male =9 Age range =57–85 years	Semistructured interviews	Qualitative: interpretive phenomenological
Skilbeck et al (1998)^45^ UK	Health district	63	Diagnosis of chronic bronchitis, emphysema, chronic asthma, pneumoconiosis, bronchiectasis, nonspecific COAD; admission in last 6 months with exacerbation for 7+ days	Male =33 Age range =55–80 years	In-depth interviews Quality of life/resource use questionnaires	Qualitative: content Quantitative: descriptive statistics
White et al (2011)^26^ UK	GP practices	163	Diagnosis of COPD Two of the following: FEV_1_ <40%, hospital admission for COPD in last 12 months, long-term oxygen therapy, corpulmonale, use of oral steroids, housebound	Male =50% Mean age =71.63	Interview study	Quantitative: statistical analysis Qualitative: thematic
Wilson et al (2008)^44^ Canada	Pulmonary outpatients	12	Diagnosis of COPD, chronic bronchitis or emphysema; hospital admission for exacerbation in last 12 months; continuous oxygen, and considered to be in last year of life	Not reported	Longitudinal semistructured interviews	Qualitative: constant comparison
Wortz et al (2012)^23^ USA	Subset of existing trial within university health science center	47	Physician diagnosis of COPD	Male =53% Mean age =68.4	In-depth interviews	Qualitative: thematic

**Abbreviations:** COAD, chronic obstructive airways disease; COPD, chronic obstructive pulmonary disease; FEV_1_, forced expiratory volume in 1 second; GP, general practitioner; ICD-10, International Classification of Disease – version 10; LTOT, long-term oxygen therapy; MRC, Medical Research Council.

**Table 4:** Domains of support need for people with COPD

Support domains	Met needs: support needs that were met	Unmet needs: shortfalls in provision where patient needs were not met	Helpful input: supportive input perceived as helpful
**Physical**			
Understanding COPD	Feeling you have an understanding of COPD^17^ Understanding the impact of COPD on lungs^15^ Understanding the severity of symptoms and prognosis associated with a diagnosis of COPD^26^	Inadequate understanding and provision of information about the nature of COPD^16^,^18^,^21^–^24^ Not understanding, or being familiar with, the terms such as COPD and emphysema^16^,^22^ Patient not being fully aware they have a diagnosis of COPD^19^ Lack of discussion about the nature of COPD^19^,^20^,^25^ Needing a greater understanding about what is happening to the lungs in the context of COPD^25^ Needing greater clarity about COPD at the time of diagnosis^16^,^18^,^19^,^27^ Not receiving enough information about prognosis and disease progression^18^,^19^,^21^,^25^,^26^,^28^,^31^,^32^ Lack of opportunity to have an in-depth discussion about prognosis with preferred health care professional^25^,^29^–^31^	Respiratory nurses providing information about the nature of illness^14^ Information sessions within pulmonary rehabilitation classes^17^ Literature on COPD^18^ Discussions with health care professionals about prognosis and end of life care (or confidence that HCPs will bring up these issues as appropriate)^30^,^33^ Conscious discussions with health care professionals of diagnosis^29^
Managing symptoms and medications	Developing an awareness of effectiveness of disease management strategies^17^,^35^ Knowing about the effective use of medication^14^,^20^	Inadequate information about management of illness^16^ Inadequate information about how to control breathlessness or panic attacks^24^,^26^ Lack of information about medication and side effects^20^,^26^ More information/support re using inhalers and medication^20^,^22^,^24^ Lack of advice re managing exacerbations^20^ Better awareness of treatment options and costs and benefits of medication^20^,^25^ Need for medication to be reviewed^25^ Lack of proactive monitoring^30^,^32^ Uncertainty about the provision of medical support after discharge^34^ More support to use standby medication effectively^34^ Lack of knowledge about provision and use of oxygen and nebulizers^34^ Help knowing what to do, or when to seek help, when symptoms deteriorate^20^,^21^,^24^,^32^,^34^ Uncertainty about who to contact during the night^34^ Someone to help make decisions about what to take or do when unwell^36^	Respiratory nurses providing information about breathing techniques and effective use of medication^14^ Pulmonary rehabilitation provided support in terms of learning to cope with symptoms/using inhalers/breathing exercises^15^,^17^,^22^ Booklets providing information about breathing exercises^24^ Specialist nurses being available over the weekends^14^ Support from GP to manage exacerbations^32^,^35^ Proactive monitoring after admission^32^ Easy access to GPs who can respond to calls for assistance^14^ Discussions with health care professionals about treatment options^29^ Guidance and feedback from health care professionals about self-management^17^,^35^ Monitoring from GP after hospital admission^34^ Having a named HCP or family who will respond to immediate concerns^14^,^15^,^32^,^37^
Healthy lifestyle	Able to discuss or address smoking behaviors^39^	Suggestions on how to change lifestyle^20^ Support to exercise/use own exercise equipment safely at home^24^ Strategies to facilitate smoking cessation/access to smoking cessation programs^38^ Opportunities to discuss lifestyle choices in a nonjudgmental context^20^	Provision of a safe environment in which to exercise provided via Pulmonary Rehabilitation classes^17^,^22^ Support and encouragement to exercise/stay active offered by physiotherapists at pulmonary rehabilitation classes^17^,^22^ Encouragement from HCP to stop smoking^38^ Provision of a nonjudgmental context^38^ Access to smoking cessation/pulmonary rehabilitation and structured home exercise program^38^ HCP providing praise and support during smoking cessation^39^
**Psychological and emotional**			
Managing feelings and worries	Ability to overcome feelings of low self-worth, sadness, and lack of confidence^40^ Able to express distressing emotions^15^,^27^,^40^	Dealing with feelings of frustration and anxiety^22^ Supporting patient psychologically and preventing pessimism^20^	HCPs providing opportunities to share feelings, be listened to, and feel understood^14^,^15^,^40^ HCPs delivering care in a way that is personalized/conveys that the patient is an individual/makes the patient feel cared for/creates a nonjudgmental context^27^,^35^,^40^ Seeing others with COPD coping^15^ Opportunities for mutual support provided by contact with peers^15^
Living positively with COPD	Overcoming feelings that you are alone in having COPD^27^ Experiencing a sense of validation of feelings and experiences^27^ Overcoming guilt and letting go of self-criticism^27^	Feeling that you are the only person with COPD^35^	Peer support provides opportunities for sharing and validating experiences with understanding others^27^,^35^ Support and encouragement to live positively with COPD^15^,^17^,^22^,^35^
Thinking about the future	Able to discuss and plan for the future: treatment, services, funeral arrangements, and financial and legal issues^19^,^21^,^26^,^31^,^33^	Opportunity to address emotions in relation to the future^31^,^40^ Information about the availability of community supports and accommodation for people in the advanced stage of illness^21^ Opportunities to discuss and plan for the future treatment and care^19^,^31^,^33^	Positive impact of meeting others facing end of life^31^,^40^
Anxiety and depression			Access to psychological support and specialist services (talking therapies)^15^,^22^
**Social**			
Practical support	Able to live at home and maintain some independence^36^	Someone to be the patients’ voice when energy is insufficient^36^	Provision of personal care by family: medication, dressing, and food and drink preparation^30^,^32^,^37^,^42^,^44^ Provision of practical help by family and friends: lifting oxygen tanks, gardening, lifts, housework, and shopping^36^,^37^,^42^–^44^ Support with personal care provided by professional carers^36^,^44^
Finance, work, and housing	Financial support facilitates ability to live in a better way^22^,^27^ Able to discuss and plan for the future: funeral arrangements and financial and legal issues^21^,^26^	Lack of information and support to access financial benefits^16^,^22^,^30^,^34^,^45^ Lack of information about housing options^21^,^34^	Support from respiratory nurses to apply for benefits^16^ Information provided at pulmonary rehabilitation sessions about accessing benefits^22^
Social and recreational life	Access to transport or assistive devices such as wheelchairs facilitates ability to participate in social activities^36^,^42^	Lack of transportation to access social and recreational support^37^	Pulmonary rehabilitation and hospice facilities provide opportunities to meet people and make friends^17^,^22^,^40^ Opportunities to participate in activities via hospice day provision^40^ Family and friends provide lifts^44^
Navigating services		Difficulty accessing and obtaining services^18^ Lack of information about available services^16^,^34^	Families and friends accompanying patients to appointment to assist with understanding, making appointments, anxiety, assimilating, and providing information^37^
Maintaining independence	Mobility and independence increased due to access to assistive devices, eg, wheelchairs^36^ Patients have access to chairlifts, bath aids, and other assistive devices^16^,^43^,^45^	Lack of equipment to promote mobility, eg, wheelchairs/stairlifts^16^,^32^,^36^,^42^,^45^ More information about, and better access to, aids and adaptions^16^,^30^,^32^,^45^	Social services provision of chairlifts^16^
Families and close relationships		Access to information about COPD for carers^22^ Access to support for carers^30^	

**Abbreviations:** COPD, chronic obstructive pulmonary disease; GP, general practitioner; HCP, health care professional.
